# Simulation Study of Process-Controlled Supramolecular Block Copolymer Phase Separation with Reversible Reaction Algorithm

**DOI:** 10.3390/polym12030528

**Published:** 2020-03-01

**Authors:** Jian-Bo Wu, Hong Liu, Zhong-Yuan Lu

**Affiliations:** 1 State Key Laboratory of Supramolecular Structure and Materials, Institute of Theoretical Chemistry, Jilin University, Changchun 130023, China; wujianbo@nxu.edu.cn; 2 State Key Laboratory of High-Efficiency Coal Utilization and Green Chemical Engineering, College of Chemistry and Chemical Engineering, Ningxia University, Yinchuan 750021, China; 3 Key Laboratory of Theoretical Chemistry of Environment Ministry of Education, School of Chemistry, South China Normal University, Guangzhou 510631, China

**Keywords:** supramolecular diblock copolymer, reversible reaction, dissipative particle dynamics

## Abstract

A supramolecular diblock copolymer formed by reversible bonds between the two blocks shows a rich microphase separation behavior and has great application potential in stimuli-responsive materials. We propose a novel method to describe supramolecular reactions in dissipative particle dynamics, which includes a reversible reaction to accurately reproduce the strength, saturation, and dynamic properties of the reversible bonds in the simulations. The thermodynamic properties and dynamic processes of the supramolecular diblock copolymer melts in both equilibrium and non-equilibrium states were studied using this method. The simulation results show that the method can faithfully characterize phase behaviors and dynamic properties of supramolecular diblock copolymer melts, especially in a non-equilibrium state, which provides a novel tool to unveil self-assembly mechanism and describe the properties of supramolecular block copolymers.

## 1. Introduction

Supramolecular block copolymers [1] are a type of block copolymers in which polymeric blocks are connected by non-covalent bonds [2]. These non-covalent bonds include multiple hydrogen bonds [3,4,5], host-guest interactions [6], donor acceptor interactions [7], metal-coordination bonds [8,9,10], and so on. Due to the reversible and dynamic nature of non-covalent bonds, supramolecular block copolymers are typically stimuli-responsive. Components in supramolecular block copolymers can be easily removed in a specific phase structure (for example, one only needs to dissolve the corresponding components to remove) [11], therefore this feature can be used to simplify the process of manufacturing nanofiltration membranes and nano-templates, or create stimuli-responsive materials [12]. A number of novel hierarchical structures have also been obtained using the reversible nature of non-covalent bonds, which can be used to prepare photovoltaic materials and integrated circuits [13,14,15]. However, it has also been found from experimental and theoretical simulations that the dynamical pathway is the key to the formation of final phase structure of supramolecular block copolymers and the elimination of its defects [16,17]. In order to achieve the control of supramolecular block copolymer phase behavior, the thermodynamic and dynamic mechanism should be clearly understood.

Many examples about the dynamical path affecting the phase structure of block copolymers have been reported in experiments [17,18,19]. For example, different orientations of block copolymer microphase separation domains could be obtained by annealing with suitable solvents [11]. Researchers also used external fields (such as electric, magnetic, flow [20], and pressure) to eliminate defects and control the formation of novel nanostructures [21]. These methods of controlling phase structures are typically carried out in a non-equilibrium condition, but theoretical and simulation studies on the microscopic mechanism of this process in supramolecular block copolymer systems are still scarce [22].

At present, the theoretical and simulation studies on supramolecular block copolymers are mainly focused on equilibrium state. Random phase approximation [23] and self-consistent field theory (SCFT) [24] were used to study the macrophase separation and microphase separation of supramolecular diblock copolymer (SDC) systems. Later, people used these methods to study a variety of SDCs: those with associating sites at the chain ends, diblock (AB + B type [25]), triblock (ABA type [26,27]), comb-like (A/B type [28], AB/A type [29]), and monomers on the chain with association sites (A/B type [30], AB/C type [31] and AB/B’-C type [32]). Monte Carlo (MC) simulation method has also been used to study the self-assembly of SDC in melt [33,34] and solution [35,36]. Lísal et al. used an extended ensemble to describe the chemical reaction process, and developed the RxDPD method and studied the SDC system in detail [37,38]. These methods are typically very successful when describing the energetics and retrieving thermodynamic stable states under given conditions. With the in-depth application of SDCs, researchers have begun to pay more attention to issues such as the dynamic effects of the system on phase structure and phase growth kinetics [22]. To this end, it requires that the simulation of SDCs must be able to accurately reproduce the dynamics of the system.

In the simulation studies of SDC gel and solution systems, people have hybridized MC with other simulation methods to describe the dynamic evolution of reversible bonds [39,40,41,42,43,44]. Hoy and Fredrickson studied the dynamic behavior of associative polymer gel systems in equilibrium [40] and non-equilibrium states [41]. Sing and Alexander-Katz studied the collapse kinetics caused by the equilibrium between connections of monomers in a single chain [42], and the stretching process in non-equilibrium conditions [43]. Inspired by hybridized molecular dynamics (MD)–MC methods, we introduce a reversible reaction algorithm to faithfully describe the strength and dynamic characteristics of reversible bonds, and incorporate it in dissipative particle dynamics (DPD) to simulate the dynamic behavior of SDC microphase separation, so that the system not only possesses the characteristics of reversible bonds but also accurately shows the phase structures and dynamics of SDCs. This algorithm is simple and easy to implement, and the thermodynamic properties of reversible bonds in SDCs can be related to the parameters of a reversible reaction model to achieve mapping from top down. It can solve some of the main dynamics problems in the phase separation of SDCs: how the system responds to changes in external conditions; how the phase structure depends on the kinetic path, and so on.

We will introduce the details of DPD simulation and the reversible reaction algorithm in Section 2; in Section 3, we will take SDCs as an example, and apply this method to study their microphase separation both in equilibrium and non-equilibrium states. Finally, conclusions are drawn in Section 4.

## 2. Simulation Model and Methods

### 2.1. Dissipative Particle Dynamics

Dissipative particle dynamics have been developed since the 1990s [45,46,47,48]. It is a mesoscopic simulation method that successfully bridges the gap between microscale and macroscale. Groot and coauthors used DPD to simulate polymer systems and successfully characterized the structural properties, especially for block copolymers [47,48]. In their simulations, one polymer chain can be mapped to a number of particles connected by a classical bonding potential, according to the number of segments in a polymer.

The mass of the i-th DPD particle is $m_{i}$, the position is $r_{i}$, and the velocity is $v_{i}$. The interaction cut-off is $r_{0}$. For simplicity, the mass of each DPD particle is taken the same and set as the unit of mass in the simulations. Similarly, the cut-off distance $r_{0}$ is set as the unit length of the system, the Boltzmann constant *k* multiplied by temperature *T* is set as the unit of energy, and for easy numerical handling, we define m=1.0, $r_{0}=1.0$, and kT=1.0, so the unit of time is $\tau_{0}=\sqrt{r_{0}m/kT}=1.0$.

The total force $F_{i}^{tot}$ on the i-th particle includes conservative force $F_{ij}^{C}$, dissipative force $F_{ij}^{D}$ and random force $F_{ij}^{R}$, i.e.,

(1) $$F_{i}^{tot}=\sum_{j}(F_{ij}^{C}+F_{ij}^{D}+F_{ij}^{R}).$$

Conservative forces $F_{ij}^{C}$ include repulsive forces $F_{ij}^{rep}$ and bonding forces $F_{ij}^{bond}$.

(2) $$F_{ij}^{rep}=\{\begin{array}{c} \alpha_{ij}\left(1-\left|r_{ij}\right|\right){\hat{r}}_{ij},\;\;if\;\left|r_{ij}\right|<1.0\\ 0,\;\;if\;\left|r_{ij}\right|>1.0 \end{array},$$

where $\alpha_{ij}$ is a maximum repulsion between particle i and particle j. $r_{ij}=r_{i}-r_{j}$ and ${\hat{r}}_{ij}=r_{ij}/\left|r_{ij}\right|$.

(3) $$F_{ij}^{bond}=K\left(\left|r_{ij}\right|-r_{e}\right){\hat{r}}_{ij},$$

where K is the spring coefficient and $r_{e}$ is the spring equilibrium length, set as K=4.0 and $r_{e}=0$, respectively. The expression of the dissipative force is

(4) $$F_{ij}^{D}=-\frac{1}{2}\sigma^{2}{\left(\omega \left(r_{ij}\right)\right)}^{2}/kT\left(v_{ij}\cdot {\hat{r}}_{ij}\right){\hat{r}}_{ij},$$

where σ is the noise amplitude, $\omega \left(r_{ij}\right)$ is weight function, $v_{ij}=v_{i}-v_{j}$; the expression of the random force is

(5) $$F_{ij}^{R}=\sigma \omega \left(r_{ij}\right){\hat{r}}_{ij}\zeta /\sqrt{\Delta t},$$

ζ is a Gaussian random number with zero mean and unit variance. Δt is the integration timestep. This regular ensemble obeys the fluctuation-dissipation theorem. The time evolution of DPD particles is determined by Newton’s equations of motion.

The Flory–Huggins theory is the simplest mean field theory for describing the thermodynamic properties of polymer blends. The theory has achieved great success in describing compatibility between different polymers and their phase behavior. Groot et al. [47,48] mapped the atomic and molecular structure information of the real polymer system to the DPD model, and defined the repulsive interaction parameter $\alpha_{ij}$ in DPD simulation by the Flory–Huggins χ parameter, making the DPD method one of the most suitable particle-based simulation methods to describe polymer phase behavior.

### 2.2. Reversible Bond Algorithm

Previous researchers used the MD–MC hybrid algorithm to simulate the reversible bond reaction in polymer solution systems [40,49,50,51]. The reversible bond reaction of the SDCs involves the following two sub-reactions:

(6) $$A-\ldots A_{i}^{*}+B_{j}^{*}\ldots -B\;\overset{k_{B}}{\Rightarrow }\;A-\ldots A_{i}-B_{j}\ldots -B,$$

(7) $$A-\ldots A_{i}-B_{j}\ldots -B\;\overset{k_{UB}}{\Rightarrow }\;A-\ldots A_{i}^{*}+B_{j}^{*}\ldots -B.$$

This reversible reaction process is schematically shown in Figure S1 (in Supplementary Materials). A particle at the chain end may react with another particle containing a complementary associative group to form a reversible bond, and the bond potential is also expressed by Equation (3). Generation and breaking of the bonds are based on the Bell reaction model [52]. $E_{B}$ and $E_{UB}$ correspond to barriers of a bonding energy profile. They represent the bonding activation energy and the unbonding activation energy, respectively. $h=E_{UB}-E_{B}$ and is determined by the energy difference between the bound and the unbound states. The larger the value of h is, the easier it is to form a bond. Based on this model, we set the probability that each reactive particle pair will bond as $Pr_{B}=\exp\left(-E_{B}/kT\right)$ and the probability that a bond will break as $Pr_{UB}=\exp\left(-E_{UB}/kT\right)$, respectively.

In a reversible reaction step, for any reactive A particle, according to its bonding situation, it can enter a bonding process or an unbonding process. If the A is not bonded, it will enter a bonding process: test the unreacted B particles within the reaction radius $r_{c}\;$ of A, and generate a unit random number R. If $R<{\mathrm{Pr}}_{B}$, create a new bond. If the A has already formed a bond, it will enter the unbonding process: a unit random number R is generated. If $R\;<{\mathrm{Pr}}_{\mathrm{UB}}$, break the bond and generate unreacted A and B particles. The reversible reaction flowchart can be seen in Figure S2 (in Supplementary Materials).

The reaction rate constants are:

(8) $$k_{B}=v_{B\;}\exp\left(\frac{-E_{B}}{kT}\right)\;,$$

(9) $$k_{UB}=v_{UB\;}\exp\left(\frac{-E_{UB}}{kT}\right).$$

Here $k_{B}$ and $k_{UB}$ are bonding and unbonding rate constants, respectively, and $v_{B}$ and $v_{UB}$ are attempt frequencies. $v_{B}$ and $v_{UB}$ depend on the period of execution of the reaction attempts ($\tau_{R}$) and the number of reaction particles within the reaction radius ($r_{c}$). In this case, the equilibrium constant is:

(10) $$K_{eq}=\frac{k_{B}}{k_{UB}}=\frac{v_{B\;}}{v_{UB\;}}\exp\left(\frac{h}{kT}\right).$$

### 2.3. Simulation Details

In this paper, we further apply the reversible bonding algorithm to study the phase separation behavior of classical supramolecular diblock copolymer systems. In the simulation, we consider the non-covalent bond between the connecting blocks as a reversible reaction. These groups that can form non-covalent bonds are reactive. We focus on the A5B5 system (consisting of homopolymers A5, a homopolymers chain B5 and diblock polymers A5-B5) and the A3B7 system (consisting of homopolymers A3, homopolymers chain B7 and diblock polymers A3-B7) with supramolecular chain length of N=10. The size of simulation box is set to $30\times 30\times 30r_{0}^{3}$. The random noise amplitude σ=3.0 and integration time step $\Delta t=0.05\tau_{0}$. The integration algorithm is modified velocity–Verlet [48]. Periodic boundary conditions are applied to all directions of the simulation box. The number density of the DPD particles in the system is ρ=3.0, so a total of 81,000 particles are included in each simulation. The fraction of the reactive particles A (B) was 0.1. The interaction parameters of the conservative force in DPD, $\alpha_{ij}$, correspond to the Flory–Huggins χ parameters:

(11) $$\alpha {\;}_{ij}=\alpha_{ii}+3.27\chi ,$$

where $\alpha_{ii}=25.0$ is the strength of the interaction between the same species in the system.

We use the GALAMOST package [53] to perform DPD simulation with the reversible reaction algorithm. In the simulation, the reaction step is executed every 20 integration time steps, i.e., $\tau_{R}=\tau_{0}$. All A* particles and reversible bonds are tested in each reaction step. The reaction radius $r_{c}$ is set as 1.25$r_{0}$. By adjusting any two of $E_{B}$*_,_* $E_{UB}$ and h in the reversible bonding model, we can realize the control of the kinetics and strength of reversible bond.

## 3. Results and Discussion

### 3.1. Dynamic Properties of the Reversible Bond

In a supramolecular diblock copolymer simulation, the extent of the reversible reaction and its kinetics can be easily controlled by the parameters of reversible reaction algorithm presented in a reactive bonding model. We chose the A5B5 system with incompatibility χN=42 as an example to show the characteristics of the algorithm.

The unbonding reaction is a first-order reaction. Therefore, it can be seen from Equation (9) that when the reversible bond strength h is constant, the bond-breaking reaction rate will be determined by the bonding reaction barrier. And

(12) $$\tau_{b}=k_{UB}^{-1}=v_{UB}^{-1}\exp(\frac{E_{B}+h}{kT})\propto \exp(\frac{E_{B}}{kT}).$$

**Figure 1:**
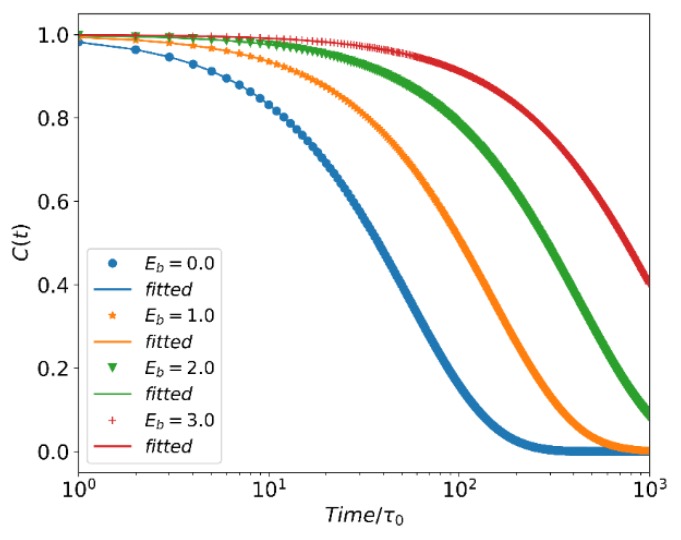
Reversible bond autocorrelation function $C_{b}\left(t\right)$ for different values of bonding activation barrier $E_{B}$.

The reversible bond lifetime, $\tau_{b}$, can be obtained from the autocorrelation function of the reversible bond, i.e.,

(13) $$C_{b}\left(t\right)=(\sum_{i,j}h_{i,j}\left(t\right)\cdot h_{i,j}\left(0\right))/\sum_{i,j}h_{i,j}\left(0\right),$$

where the $h_{i,j}\left(t\right)$ value is determined to be 1 or 0 depending on whether or not the active chain end particles $A_{i}^{*}$ and $B_{j}^{*}$ are bonded at that time t. The $h_{i,j}\left(t\right)$ of each bond is only allowed to be counted once. $C_{b}\left(t\right)$ versus t is shown in Figure 1 for different bond reaction barriers when h=4. We find that the autocorrelation function decays in a simple exponential form:

(14) $$C_{b}\left(t\right)=\exp\left(-t/\tau_{b}\right).$$

It indicates that the generation and breaking of the reversible bond is completely a random walk process.

**Figure 2:**
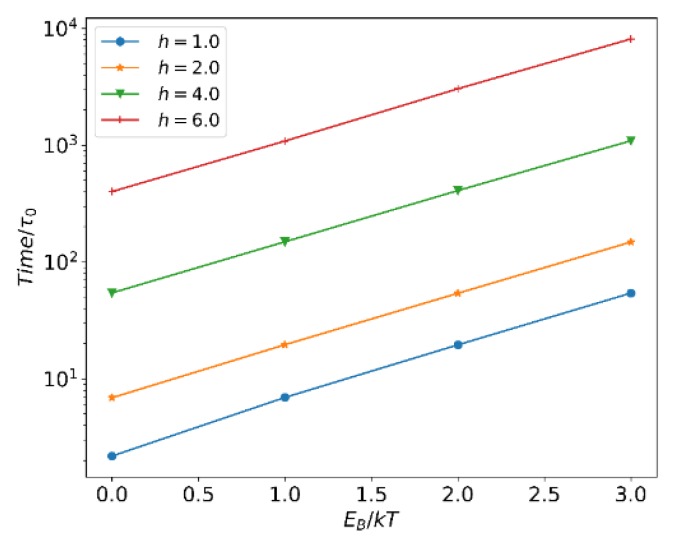
Dependence of the reversible bond life time $\tau_{b}$ on $E_{B}$.

Figure 2 shows $\tau_{b}$ versus $E_{B}$ of the reversible bond at different h. As expected, $\tau_{b}$ and $E_{B}$ confirm Equation (12). The results prove that the reversible bond algorithm can accurately reflect the dynamic characteristics of the reversible reaction. Thus we can easily adjust the lifetime of the reversible bond by controlling $E_{B}$ in the simulations.

**Figure 3:**
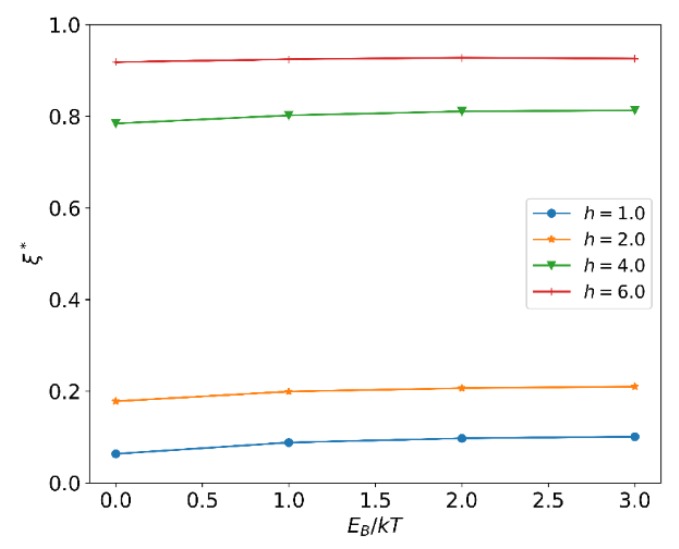
The normalized extent of reaction $\xi^{*}$ at different $E_{B}$.

When the reversible bond strength h is constant, does the reaction rate affect the equilibrium composition of SDC? We then calculate the normalized extent of reaction $\xi^{*}$ at different $E_{B}$. The results are shown in Figure 3. It can be seen that the change of $E_{B}$ does not affect the reaction progress. It shows that equilibrium composition of the system is determined by the energy difference h, and the bond lifetime takes no effect on equilibrium composition of SDC. This is because the reaction period is sufficiently long, so that the free particles from the bond-breaking reaction have enough time to diffuse out of the reaction radius. Such a result is consistent with the self-consistent field theory model of the SDC [33] and the assumption in the RxDPD [38].

In DPD simulation, a bead can represent multiple monomers to extend length and time scale of the simulation. In our simulation, a reactive DPD bead represents several unreactive monomers and a reactive terminal monomer. This makes both the time scale and space scale of a coarse-grained simulation larger than a fine-grained (monomer scale), so our simulation can truly reflect which bond lifetime is longer than the minimum simulation time scale. For the bond lifetime less than the minimum simulation time scale, the dynamic effects of simulation cannot be characterized. In the latter case, the number of monomers represented by a DPD bead, the simulation time step, and the reaction period ($\tau_{R}$) can be reduced to make the bond lifetime less than or equal to the minimum time scale of the simulation.

Previous studies of gels [40,54] and vitrimers [55] have shown that the reversible reaction rate is a key factor affecting the relaxation of the entire system. For self-assembly of SDCs, the reversible reaction rate affects the phase separation and the self-assembly rates. For most SDCs, the reaction rate can be changed by varying the temperature and the type of functional groups. For some dynamic covalent bonds, the reaction rate can be conveniently adjusted by changing the type or dosage of the catalyst. However, the dynamic properties of the reversible bond have an effect on the dynamic properties of the system, such as the viscosity, which we will further demonstrate in Section 3.4. Next, we focus on the effects of h and χN on the phase separation behavior of the system.

### 3.2. The Effect of Reversible Bond Strength h on SDC Phase Behavior

To investigate the effect of reversible bond strength on the phase behavior of the system, we fix χN=42 and change reversible bond strength with an increment/decrement of Δh=±0.1kT per $5000\tau_{0}$. In order to clearly indicate the phase structure of the SDC system, we introduce the order parameter of particle j (j=A, B), which can be expressed as

(15) $$O_{2,j}=\frac{1}{V}\int_{V}\left[\rho_{j}^{2}\left(r\right)-\rho_{j}^{2}\right]dV.$$

where $\rho_{j}\left(r\right)$ is the local density of particle *j*, which can be approximated as the number density in a sphere of radius 1.25*r*_0_, and $\rho_{j}$ is the overall density. The averaged order parameter $O_{2}=\left(O_{2,A}+O_{2,B}\right)/2$ is used in this paper. With this definition, integration of Equation (15) results in

(16) $$O_{2}^{max}=f_{A}f_{B}\rho^{2},$$

where $O_{2}^{max}$ corresponds to ideal separation of A and B particles into two separating domains, $f_{A}$ ($f_{B})$ is the total volume fraction of particles A (B).

**Figure 4:**
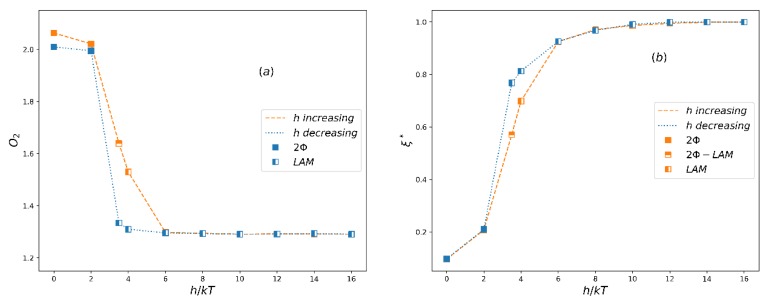
(**a**) The averaged order parameter, $O_{2}$, and (**b**) the normalized extent of reaction, $\xi^{*}$, as a function of the reversible bond strength, *h*, for system A5B5. LAM denotes a lamellar phase, 2Φ-LAM denotes a coexistent phase and 2Φ denotes a two-phase state.

**Figure 5:**
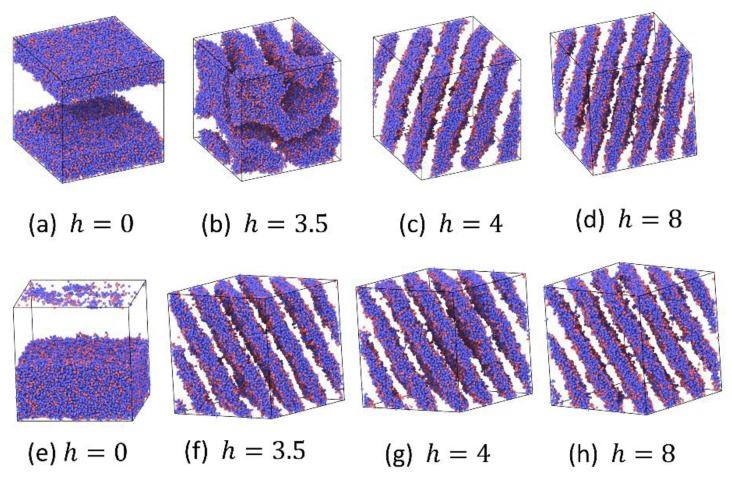
Snapshots of system A5B5 at different values of the reversible bond strength, h. Increasing h process: (**a**–**d**); decreasing h process: (**e**–**h**). Red color denotes reactive A particles and blue color denotes nonreactive A particles. For clarity, B particles are not shown. All following phase separation morphology snapshots use the same settings.

The order parameters for the A5B5 system in both increasing h process starting from smaller h (corresponding to weaker bond) to larger h (corresponding to stronger bond) and decreasing h process running oppositely are shown in Figure 4a. As expected, the order parameter *O*_2_ is close to the theoretical value $O_{2}^{max}=2.25$ when h≤2, which corresponds to macrophase separation (i.e., two-phase, 2Φ), and when h≥4, the *O*_2_ value is about 1.3, which corresponds to the lamellar phase (LAM) in microphase separation. At the same time, an apparent hysteresis between increasing and decreasing h processes occurs in the phase coexistence region (2Φ-LAM). The normalized extent of reaction in this process (Figure 4b) is also consistent with the change of *O*_2_. Figure 5 shows the morphology of typical phases in this process. It can be seen that the domain size of the lamellar phase at h=4 is larger than that at h=8. This is mainly because the extent of reaction is small when h is small, and more homopolymers swell the lamellar structure.

**Figure 6:**
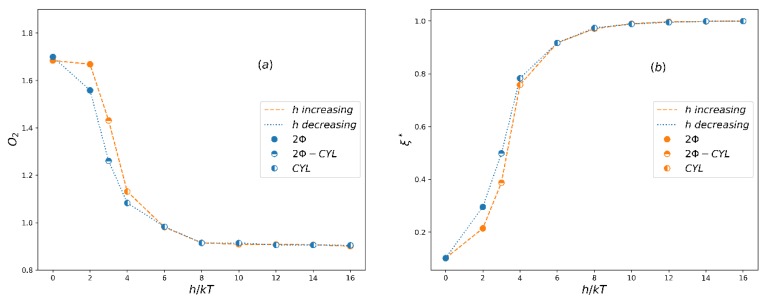
(**a**) The averaged order parameter, *O*_2_, and (**b**) the normalized extent of reaction, $\xi^{*}$, as a function of the reversible bond strength, h, for system A3B7. CYL denotes a cylindrical phase, 2Φ-CYL denotes a coexistent phase and 2Φ denotes a two-phase state.

**Figure 7:**
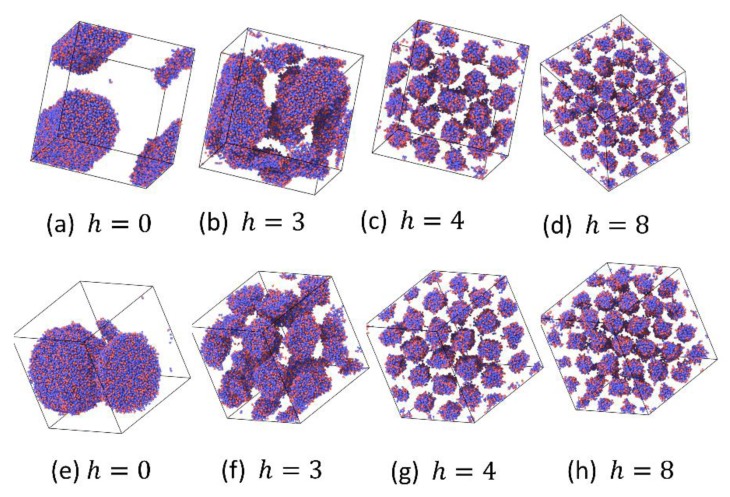
Snapshots of system A3B7 at different values of the reversible bond strength, h. Increasing h process: (**a**–**d**); decreasing h process: (**e**–**h**).

In order to further verify the reversible bond algorithm, we also examine the order parameters, extent of reaction and phase separation morphology of the A3B7 system by increasing and decreasing h similar to that in A5B5 systems. The results are shown in Figure 6 and Figure 7. When the reversible bond strength of the the A3B7 system is very weak, it stays in 2Φ state, and *O*_2_ also reaches the maximum value, which is very close to the theoretical maximum value of 1.89. When the cylindrical columnar phase (CYL) is formed, the *O*_2_ value is in the range of 0.85~1.2. The normalized extent of reaction (Figure 6b) and morphology (Figure 7) also show that the domain size of the columnar phase decreases with increasing h. In the 2Φ-CYL phase region, a hysteresis also appears, but the hysteresis is weaker than that in A5B5 system. It reflects the fact that at the same thermal energy input (kT=1.0), 2D continuous LAM structure is more difficult to change as compared to 1D continuous CYL structure.

Experimentally, for SDCs formed by using multiple hydrogen bonds or donor-acceptors, the reversible bond strength can be regulated by changing temperature [56], pH value [57], ion concentration [10] and so on. New types of stimuli-responsive materials and sensors have been designed using the sensitivity of material phase transitions to the strength of reversible bonds. The phase hysteresis in this process is detrimental, especially where low latency is required. However, for the preparation of special morphology, phase hysteresis can be used to expand the range of processing parameters [16]. Our reversible bond algorithm can accurately simulate this phenomenon, which can be applied to design functional materials and studies on the mechanism.

**Figure 8:**
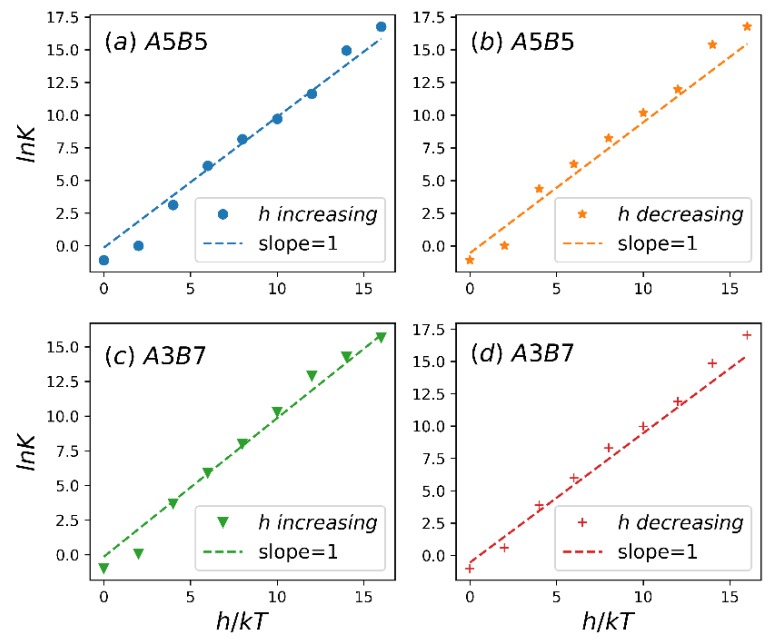
The logarithm of equilibrium constant, lnK, versus the reversible bond strength, h. A5B5 system in h increasing process (**a**) and h decreasing process (**b**). A3B7 system in h increasing process (**c**) and h decreasing process (**d**).

It is possible to use the spectroscopy to obtain the bonding/unbonding equilibrium constants of some reversible bonds (hydrogen bonds, coordination bonds, etc.) [58,59]. Coleman et al. successfully used these equilibrium constants in a model for SDCs [59]. Therefore, it is very important to map the reversible bond’s equilibrium constant to a simulation parameter. In this study, we solve the reaction equilibrium condition $\mu_{1}+\mu_{2}=\mu_{3}$ under ideal-gas (IG) assumption: $\beta \mu_{i}\equiv \beta \mu_{i}^{IG}=\beta \mu_{i}^{0}+\ln\left[N_{i}/V\rho \right]$. When we assume that the DPD fluid is an ideal gas, the chemical potential of each component ($\mu_{i}$) is only related to volume fraction of the component ($N_{i}/V\rho$). Lísal adopted IG assumption to obtain the free energy of reversible bonds in the RxDPD model [38]. As a result, we obtain $N_{1}^{IG}=N_{1}^{0}-\xi^{IG}$, $N_{2}^{IG}=N_{2}^{0}-\xi^{IG}$, $N_{3}^{IG}=N_{3}^{0}+\xi^{IG}$, where $N_{1}^{0}$, $N_{2}^{0}$ and $N_{3}^{0}$ are the initial number of homopolymers and diblock copolymers, respectively, and $\xi^{IG}$ is the extent of reaction. The reversible bond equilibrium constant,

(17) $$K=\frac{V\left(N_{3}^{0}+\xi^{IG}\right)}{\left(N_{1}^{0}-\xi^{IG}\right)\left(N_{2}^{0}-\xi^{IG}\right)},$$

with a reference state at unit volume fraction. In our model, lnK=h+constant can be obtained from Equation (10). Figure 8 shows lnK plotted as a function of h, demonstrating that the simulation data fall on the line with a slope of 1. This indicates that our algorithm can match the macroscopic measurable K with h.

Since we are simulating the melt of supramolecular block copolymer, the system temperature is generally 400~500K. In the simulations, we set h range of 0~16kT, i.e., the energy corresponding to the real system is 0~80kJ/mol. This value is consistent with the bond energy range of non-covalent bonds.

### 3.3. Influence of Incompatibility Parameter χN on SDC Phase Behavior

The phase behavior of SDC is strongly influenced by the incompatibility between different blocks, which is the same as that of block copolymers. Here we fix h=5.5 and investigate the phase behavior by step-changing χN with an increment/decrement of ΔχN=±0.306.

**Figure 9:**
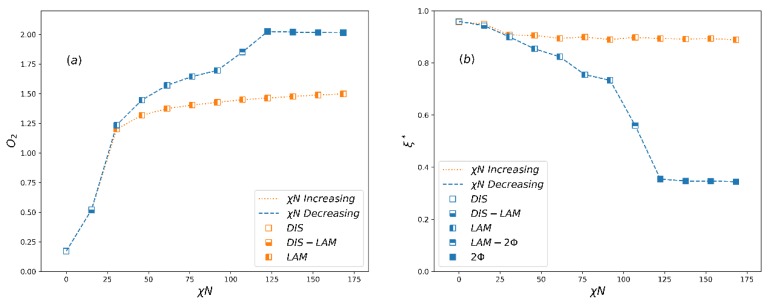
(**a**) The averaged order parameter, *O*_2_, and (**b**) the normalized extent of reaction, $\xi^{*}$, as a function of χN, for A5B5 SDC systems. DIS denotes a disordered state, DIS-LAM denotes a coexistent phase.

In the process of increasing and decreasing incompatibility, the change of $O_{2}$ in the A5B5 SDC system is shown in Figure 9a. When χN≤35, the order parameter values are small, corresponding to the disordered state. Unlike 2Φ to ordered phase transition, we find that the phase hysteresis is weak in the disordered to LAM phase transition. This is also different from block copolymers which have strong hysteresis in microphase separation [60]. This phenomenon can be mainly attributed to the acceleration of phase transition by the fast dynamics of reversible bonds. It is obvious from Figure 9 that the increasing and decreasing χN processes show great differences when χN is large. In an increasing χN process, the SDC system stays in LAM phase, and it can be seen from Figure 9b that $\xi^{*}$ remains unchanged with the increase of incompatibility. In a decreasing χN process, the A5B5 system changes from 2Φ to the LAM phase. During these two processes, the snapshots of the system A5B5 are shown in Figure S3 (in Supplementary Materials).

The A5B5 SDC system, in the process of increasing χN, starts from the disordered state (the *O*_2_ value is very small) and maintains the LAM phase after the disordered-LAM phase transition (*O*_2_ value remains at 1.2 afterwards). In the process of decreasing χN, the system starts from 2Φ phase (*O*_2_ value is close to 2.25), undergoes a 2Φ-LAM transition, and then a LAM-disordered phase transition takes place. The above results can also correspond to the normalized reaction progress (Figure 9b). The phase transition of LAM-2Φ does not occur during increasing χN, because the phase transition of LAM-2Φ has a large free energy barrier, and it is difficult to cross this barrier with the current thermal fluctuation (kT=1.0). On the contrary, the phase transition 2Φ-LAM can occur, indicating that the free energy barrier of this process is comparatively low.

**Figure 10:**
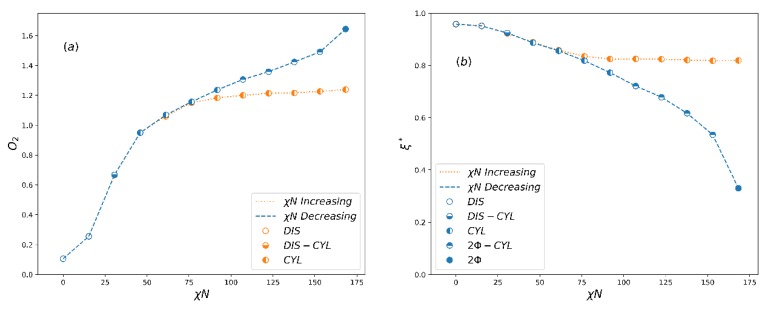
(**a**) The averaged order parameter, *O*_2_, and (**b**) the normalized extent of reaction, $\xi^{*}$, as a function of χN, for A3B7 SDC system. DIS-CYL denotes a coexistent phase.

For the A3B7 SDC system, the order parameters, normalized extent of reaction and phase separation morphology in increasing and decreasing χN processes are shown in Figure 10 and Figure S4 (in Supplementary Materials). The results are similar to those in the A5B5 system: only the disordered-CYL phase transition takes place in the increasing χN process, and the 2Φ-CYL phase transition is followed by the CYL-disordered phase transition during the decreasing χN process. However, the χN variation in 2Φ-CYL phase transition is gentler than in 2Φ-LAM phase transition. It shows that the phase transition barrier of the asymmetric system is lower than that of the symmetric system. It may be due to good solubility of component A3 compared to A5. It can promote the reversible reaction even with larger incompatibility. At the same time, the phase hysteresis in the disordered-ordered phase transition is also very small.

These results show the complex path-dependent phase separation behavior of SDC systems. It emphasizes that the phase transition process coupled with reaction is path dependent. The above simulation results show that our reversible bond algorithm can simulate the path dependence of phase behavior caused by changing incompatibility. It provides a practical simulation method for the study of the stimuli-responsive process of SDCs.

### 3.4. Non-Equilibrium Dynamics

The rheological properties of SDCs in a non-equilibrium state are important in their fabrication process (injection molding, extrusion molding, etc.) and the elimination of phase defects. In the following, we take the symmetrical A5B5 SDC system as an example and use the oscillation shear method to explore rheological properties of SDC.

**Figure 11:**
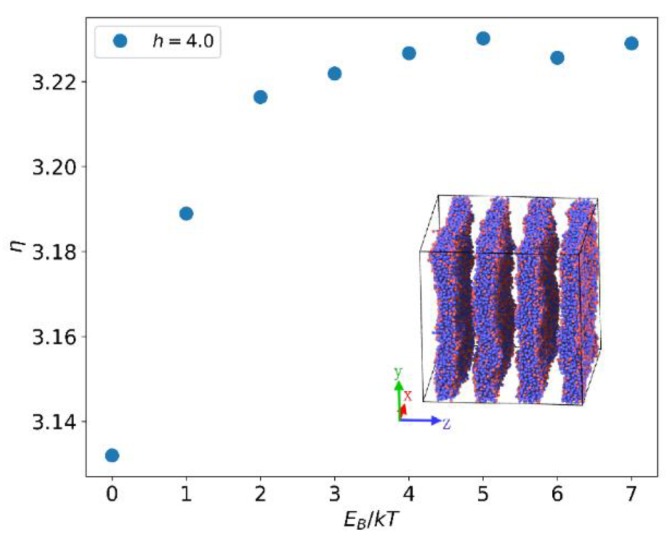
Dependence of the viscosity η on $E_{B}$. Inset shows a perpendicular lamellar structure with the gradient of the shear flow in x direction.

In the shearing process of the symmetric block copolymer, researchers found that the perpendicular orientation of the lamellae (shown in the inset of Figure 11) is more stable than the parallel orientation [61]. We use the sinusoidal external force field F=mΓsin(ωy) along the x-axis to achieve oscillatory shear, where $\omega =2\pi /L_{y}$. Γ is the maximum amplitude of force (Γ=0.1) and $L_{y}$ is the simulation box length along the y-axis. Under shearing, the velocity distribution along the y-axis has a sinusoidal function. Its viscosity is calculated by the formula

(18) $$\eta =\frac{\Gamma }{\bar{v}}\frac{\rho }{\omega^{2}},$$

where $\bar{v}$ is obtained by averaging v(t) at different times. $v\left(t\right)=2\overset{N}{\underset{i=1}{\sum }}m_{i}v_{i,x}\left(t\right)\cos\left(\omega r_{i,y}\left(t\right)\right)/\overset{N}{\underset{i=1}{\sum }}m_{i}$, where $v_{i,x}$ is the x-component of the velocity, and $r_{i,y}$ is the y-coordinate of particle i.

**Figure 12:**
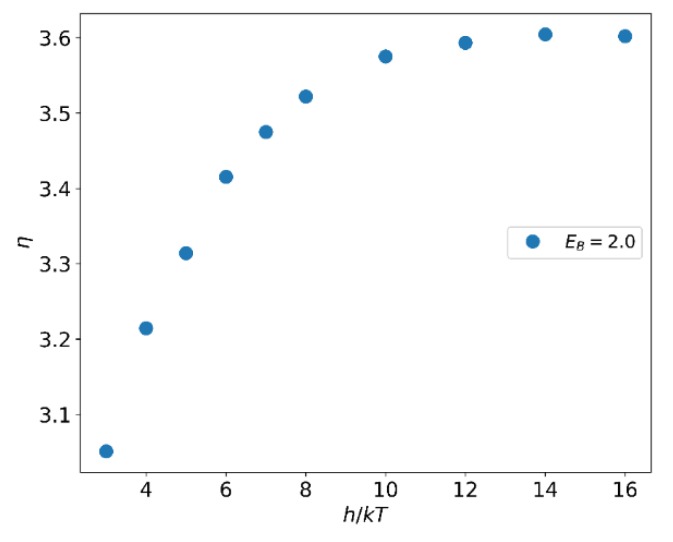
Dependence of the viscosity η on the reversible bond strength, h.

At fixed h=4.0, with increasing $E_{B}$, the viscosity of the system increases. When $E_{B}>4.0$ almost no change in viscosity is observed in the system (Figure 11). When $E_{B}$ is constant, the relationship between the viscosity of the system and the reversible bond strength is shown in Figure 12. Similar to the relationship between $E_{B}$ and viscosity, the viscosity of the system increases as the reversible bond strength h increases, and the viscosity reaches a plateau value for h>10.0.

According to Equation (12), we know that for larger $E_{B}$ or h, the lifetime of the reversible bond increases. When the total number of reversible bonds is constant, an increase in the lifetime of the reversible bond will slow the propagation of momentum along the y-axis, causing the viscosity to rise. But this effect tends to a limit after the reversible bond lifetime is greater than the shear perturbation duration. The increase in the reversible bond strength will also result in an increase in the number of bonds. It is known from Equation (17) that the increase in the number of bonds is non-linear and the increase is very limited at h>10.0.

It should be noted that, when the SDC is in a melt state, the smaller viscosity is advantageous for the SDC to undergo a phase transition or to eliminate phase defects. Our algorithm can be extended to the applications of volume expansion, external field induction, and other non-equilibrium simulations, which have a great application potential.

## 4. Conclusions

In summary, we use DPD with the reversible bond algorithm to realize SDC phase separation dynamics simulation both in equilibrium and non-equilibrium states. The simulation results show that the phase of the SDC melts depends on the reaction process. The phase behavior obtained by using this method is consistent with the results reported by using SCFT and RxDPD. By adjusting the algorithm parameters, one can easily change the kinetics and extent of the reversible reaction. Simulation results show that the reaction rate does not affect the composition and structure of SDC. However, when h or χN is changed, the phase transition takes place depending on the reaction process. In particular, there is a clear phase transition hysteresis in the transition between 2Φ and ordered phase, and this phenomenon is weaker in the disordered–ordered phase transition. In the simulations in non-equilibrium state, we get the relationship between the viscosity of the lamellar phase of SDC and the reversible bonding dynamics. By using the mapping between the reversible bond algorithm parameters and the macroscopic properties of the SDC system, one can easily map the real SDC system from top down. This method has great application potential in further research of supramolecular block copolymers.

## Supplementary Materials

The following are available online at https://www.mdpi.com/2073-4360/12/3/528/s1, Figure S1: Reaction scheme, Figure S2: Flowchart of the reversible reaction process. Figure S3: Snapshots of A5B5 SDC systems with *h* = 5.5 and Figure S4: Snapshots of A3B7 SDC systems with *h* = 5.5.
