# Supplementary material for: Simulation Study of Process-Controlled Supramolecular Block Copolymer Phase Separation with Reversible Reaction Algorithm
Source: Polymers (Basel). 2020 Mar 1;12(3):528. doi: 10.3390/polym12030528 (PMC7182871; doi:10.3390/polym12030528)
Supplement: Supplementary file 1 [file polymers-12-00528-s001.pdf]

Article

Simulation Study of Process-controlled Supramolecular Block Copolymer Phase Separation with Reversible Reaction Algorithm

Jian-Bo Wu [1,2], Hong Liu [3,*] and Zhong-Yuan Lu [1,*]

1 State Key Laboratory of Supramolecular Structure and Materials, Institute of Theoretical Chemistry, Jilin University, Changchun 130023, China; wujianbo@nxu.edu.cn (J.W.); luzhy@jlu.edu.cn (Z.L.)
2 State Key Laboratory of High-efficiency Coal Utilization and Green Chemical Engineering, College of Chemistry and Chemical Engineering, Ningxia University, Yinchuan 750021, China; wujianbo@nxu.edu.cn (J.W.)
3 Key Laboratory of Theoretical Chemistry of Environment Ministry of Education, School of Chemistry, South China Normal University, Guangzhou 510631, China; hongliu@m.scnu.edu.cn (H.L.)
* Correspondence: hongliu@m.scnu.edu.cn (H.L.); luzhy@jlu.edu.cn (Z.L.)

Supplementary information

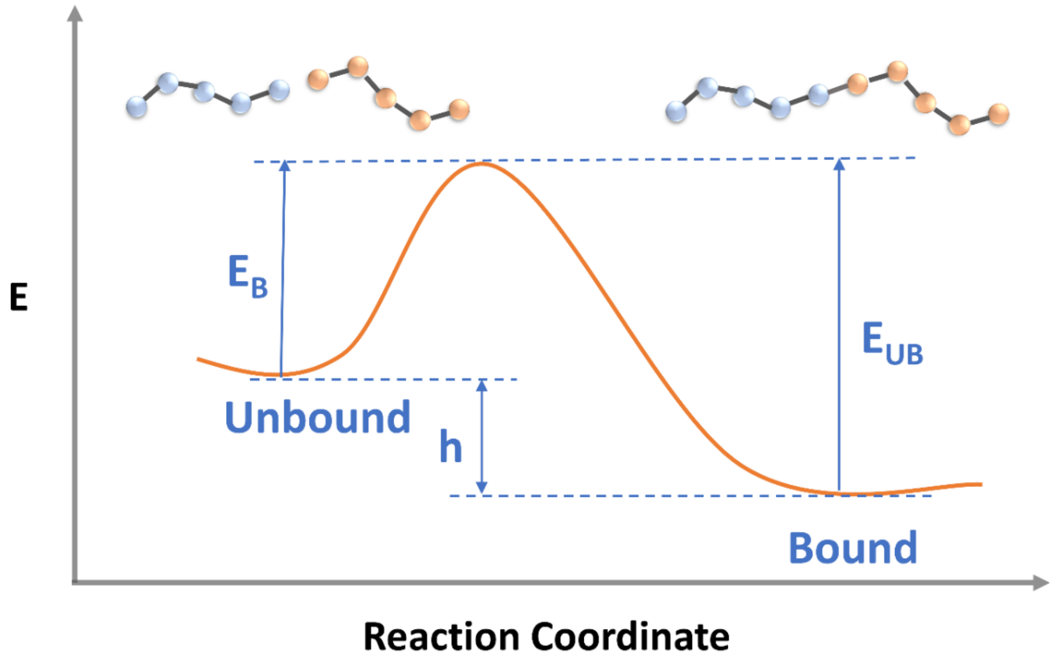

Figure S1. Reaction scheme based on the "Bell model" to represent equilibrium between two reactive particles of homopolymers in computer simulations.

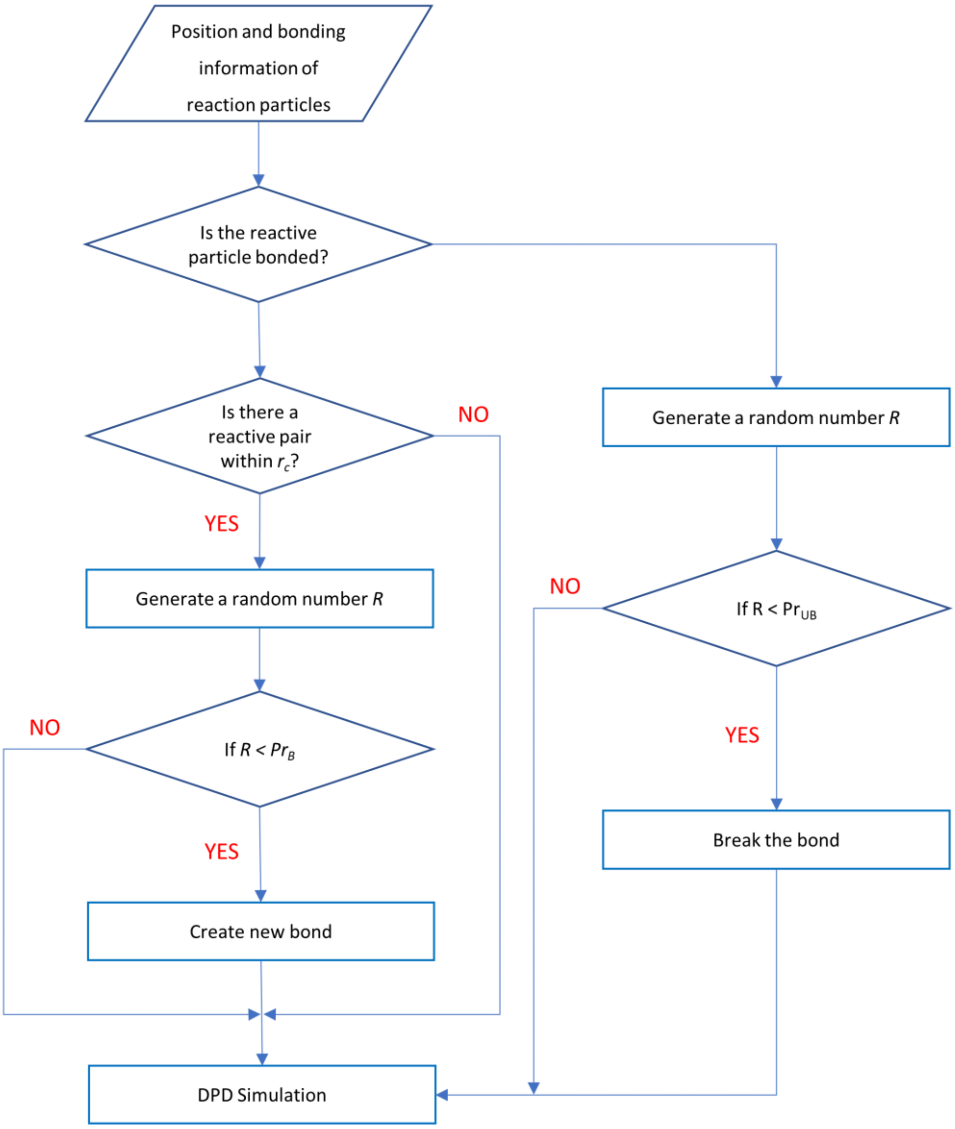

Figure S2. Flowchart of the reversible reaction process.

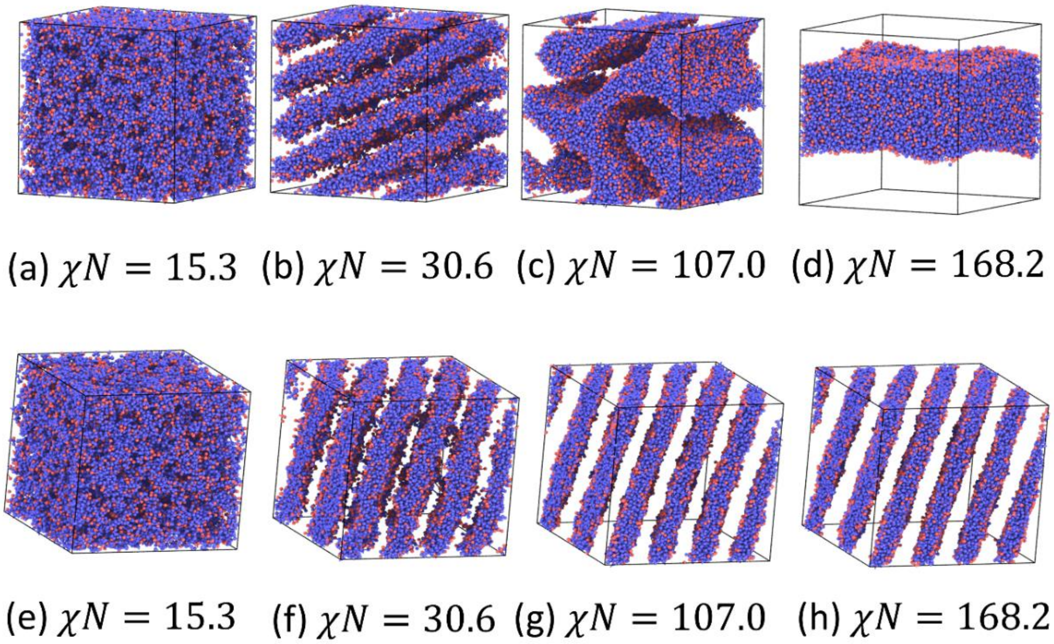

Figure S3. Snapshots of A5B5 SDC systems with $h = 5.5$ at different values of χN. Increasing χN process: (a), (b), (c) and (d); decreasing χN process: (e), (f), (g) and (h).

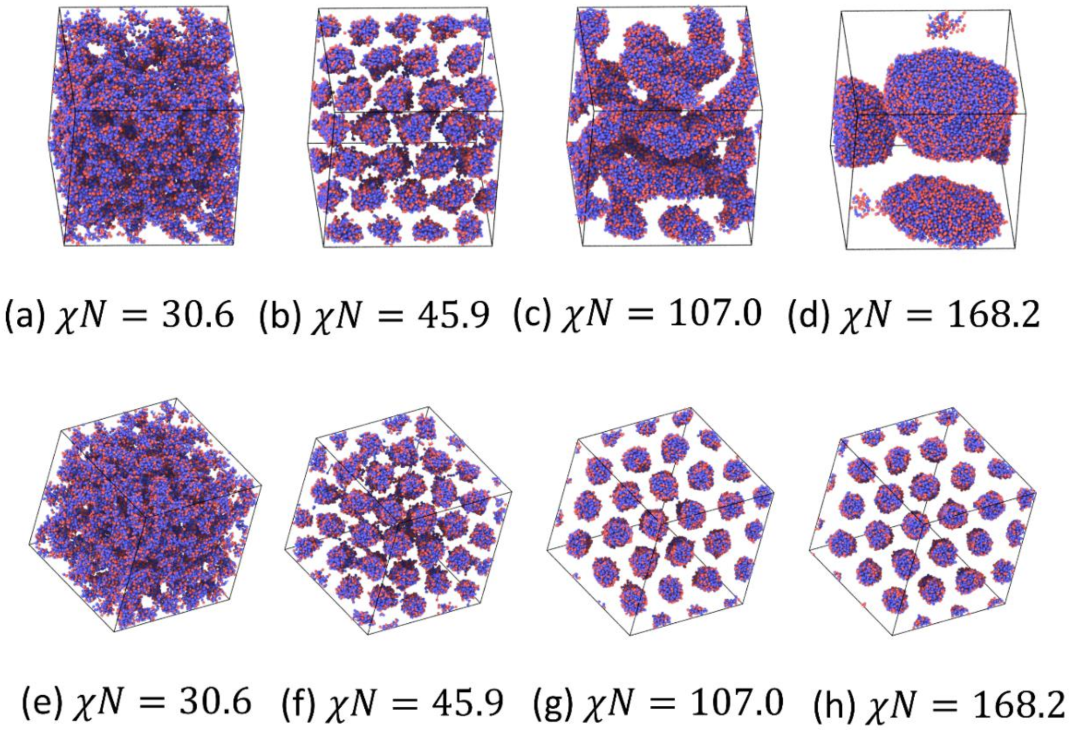

Figure S4. Snapshots of A3B7 SDC system with $h = 5.5$ at different χN. Increasing χN process: (a), (b), (c) and (d); decreasing χN process: (e), (f), (g) and (h).
